# Starch–protein interaction effects on lipid metabolism and gut microbes in host

**DOI:** 10.3389/fnut.2022.1018026

**Published:** 2022-11-16

**Authors:** Kaijun Wang, Miao Zhou, Xinyu Gong, Yuqiao Zhou, Jiayi Chen, Jie Ma, Peihua Zhang

**Affiliations:** ^1^Animal Nutritional Genome and Germplasm Innovation Research Center, College of Animal Science and Technology, Hunan Agricultural University, Changsha, Hunan, China; ^2^College of Animal Science and Technology, State Key Laboratory for Conservation and Utilization of Subtropical Agro-Bioresources, Guangxi University, Nanning, Guangxi, China; ^3^Academician Workstation, Changsha Medical University, Changsha, Hunan, China

**Keywords:** starch, protein, lipid, gut, microbes

## Abstract

The purpose of this experiment was to investigate the effects of different starch and protein levels on lipid metabolism and gut microbes in mice of different genders. A total of 160 male mice were randomly assigned to sixteen groups and fed a 4 × 4 Latin square design with dietary protein concentrations of 16, 18, 20, and 22%, and starch concentrations of 50, 52, 54, and 56%, respectively. The results of the study showed that different proportions of starch and protein had obvious effects on the liver index of mice, and there was a significant interaction between starch and protein on the liver index (*p* = 0.005). Compared with other protein ratio diets, 18% protein diet significantly increased the serum TBA concentration of mice (*p* < 0.001), and different starch ratio diets had no effect on serum TBA concentration (*p* = 0.442). It was proved from the results of ileal tissue HE staining that the low protein diet and the low starch diet were more favorable. There was a significant interaction between diets with different starch and protein levels on *Bacteroidetes*, *Firmicutes* and *Proteobacteria* abundance in feces of mice (*p* < 0.001). Compared with 16 and 18% protein ratio diets, both 20 and 22% protein diets significantly decreased the *Parabacteroides* and *Alistipes* abundance in feces of mice (*p* < 0.05), and 52% starch ratio diet significantly decreased the *Parabacteroides* and *Alistipes* abundance than 50% starch ratio diet of mice (*p* < 0.05). There was a significant interaction between diets with different starch and protein levels on *Parabacteroides* (*p* = 0.014) and *Alistipes* (*p* = 0.001) abundance in feces of mice. Taken together, our results suggest that a low protein and starch diet can alter lipid metabolism and gut microbes in mice.

## Introduction

According to the World Health Organization, more than 1 billion people worldwide are obese—650 million adults in 2022, which represents a health risk (1). Obesity is a nutritional disorder caused by an imbalance between energy intake and expenditure (2). Over the last few decades, obesity, cardiovascular disease, and type 2 diabetes have been on the rise worldwide, in order to prevent those diseases, numerous studies have attempted to reduce starch digestion and glycemic index through human diets (3–5). Recent years have seen a growing interest in developing healthy food ingredients that may enhance their protein, fiber, and nutraceutical qualities (6). Therefore, macro- and micronutrients with high participation in the diet, such as starch and protein, need to be studied and in order to develop functional foods, nutritional value and technological function had to be balanced correctly (7–9).

Historically, starch has been a major source of carbohydrates in human diets, which was absorbed as monosaccharides in the digestive tract, different functions were performed by foods depending on their chemical properties, their susceptibility to amylase, and their rate of glucose release and absorption in the gastrointestinal tract (10, 11). In order to maintain a high level of protein, new raw materials, such as soya, amaranth grain, or buckwheat, have been used as well as improved the final product’s nutritional quality and functional properties (12). Additionally, it may reduce the glycemic index (GI) of starch-based foods and prevent diabetic complications. By obstructing enzyme binding sites, protein interferes with nutritional properties and facilitates starch malabsorption in the study on starch hydrolysis (13). Animals depended on dietary protein to function physiologically. The benefits of low-protein diets in terms of resource conservation and reducing nitrogen emissions have received considerable attention in recent years. Researchers have found that reducing dietary protein levels by 3 percentage points can enhance lipid metabolism in skeletal muscle without affecting growth in pigs, while the performance of growth would be adversely affected by protein reduction of exceeding 4 percentage points (14, 15). There was ample evidence that optimum nitrogen utilization for protein accretion can be achieved by reducing protein intake in the diet and simultaneously supplementing crystalline amino acids (16).

To coordinate body health, the host developed a huge microbiota at birth. The abundance of microbes also changed and played different roles at different stages (17, 18). A better understanding of the interactions between host and gut microbes was crucial to study the complex relationship between host and microbiota. The molecules involved in this interaction could be measured, especially the microbiota produced metabolites that were available to the host. Animal health depended on gut microbiota, which were involved in digestion, metabolism, immunity, and defense against pathogens (19–21). Diet, environment, and age all influenced gut microbiota composition and activity, but diet played the largest role among them (22–24). Besides affecting the composition of the gut microbiota, diet also influenced the state of the immune system (25).

Starch and protein were thermodynamically incompatible to form complexes, but the interaction between them can also affect their respective physicochemical property (26, 27). Protein can also interact with lipid through hydrophobic or electrostatic interaction to affect their property (28, 29). These various binary interactions have been studied extensively, but to gain a better understanding of factors that can affect the quality of food product interaction between starch, protein and lipid need to be examined in detail (27, 30). There was currently little knowledge of how a low-protein diet affected the gut micro-environment and how it can solve environmental problems. The purpose of this study was to investigate the effect of different dietary protein and starch levels on lipid metabolism in mice. Moreover, we examined whether improvements in lipid metabolism also affected intestinal microbiota at different proteins and starches levels. Using male mice as a model, it was possible to systematically study the importance of different protein and starch levels on fat metabolism in mice, and to evaluate the possible harmful or beneficial effect of different levels of these two substances on the structure of the gastrointestinal tract.

## Materials and methods

### Animals and dietary treatments

This experiment was approved by the Hunan Agricultural University Institutional Animal Care and Use Committee (202105). The male C57 mice were 4 weeks old and purchased from SLAC Laboratory Animal Central (Changsha, China). The mice were housed in a controlled environment (temperature: 25 ± 2°C, relative humidity: 45–60%, and a 12-h light–dark cycle) after one week adaptation period, with free access to food and water during the experiment. The diet of mice was mainly composed of corn, soybean meal, beer yeast, casein and lard. And the diets used in the experiment as described in Table 1. The experiment lasted for four weeks.

**TABLE 1:** Composition and nutrients levels of the diet.

Items	CP:TS (16:50)	CP:TS (16:52)	CP:TS (16:54)	CP:TS (16:56)	CP:TS (18:50)	CP:TS (18:52)	CP:TS (18:54)	CP:TS (18:56)	CP:TS (20:50)	CP:TS (20:52)	CP:TS (20:54)	CP:TS (20:56)	CP:TS (22:50)	CP:TS (22:52)	CP:TS (22:54)	CP:TS (22:56)
**Ingredients**																
Corn	73.5	72	65	53.5	65.5	53	49	40	72.5	68	57	41.5	64	54	43.5	33.5
Corn starch	1	3	8	13.5	5	12.4	15	18	1.5	3	9	15.5	4.5	9	14	18
Bran	2	3.5	6	12	4	8.6	11	17.5	1	5	10	18	4	9.5	15	21
Casein	5	5	5	5	5	5.5	5.5	5	8.5	10	10	10	11	11	11	11
Soybean meal	6.5	6.5	7	7	12	11.5	11.5	11.5	9	6	6	6	9	9	9	9
Fish meal	4	4	4	4	4	4	4	4	4	4	4	4	4	4	4	4
Lard	6	4	2	2	2.5	3	2	2	1.5	2	2	3	1.5	1.5	1.5	1.5
Soybean oil	1	1	2	2	1	1	1	1	1	1	1	1	1	1	1	1
Premixture	1	1	1	1	1	1	1	1	1	1	1	1	1	1	1	1
Total	100	100	100	100	100	100	100	100	100	100	100	100	100	100	100	100
**Nutrient levels**																
GE	4405	4298	4238	4222	4260	4270	4212	4193	4256	4279	4263	4294	4291	4278	4264	4252
CP	16.1	16.2	16.3	16.3	18.2	18.1	18.2	18.0	20.1	20.3	20.2	20.2	22.1	22.1	22.1	22.2
TS	50.4	52.3	54.1	56.4	50.3	52.4	54.0	56.1	50.0	52.0	54.2	56.3	50.1	52.0	54.1	56.0

CP, crude protein; TS, starch.

A total of 160 male mice (13. 85 ± 0.27 g) were randomly divided into 16 groups with 10 repetitions in each group. Mice were fed a 4 × 4 Latin square design with dietary protein concentrations of 16, 18, 20, and 22%, and starch concentrations of 50, 52, 54, and 56%, respectively. Mice were weighed on a weekly basis. Fecal samples were obtained and kept at –80°C for further examination. The 160 mice were sacrificed by cervical dislocation with 1% pentobarbital sodium (50 mg/kg) anesthesia, and every effort was made to minimize suffering. Finally, blood, abdominal adipose tissue (AAT), liver, ileal tissue, and feces were collected for further examination.

### Analysis of biochemical parameters in blood samples

Serum extracted form blood samples using 845 rcf (g) for 10 min at 4°C. The total bile acid (TBA), total cholesterol (TC), high density lipoprotein (HDL), low density lipoprotein (LDL), triglycerides (TG), and glucose (GLU) were detected according to the standard protocol by an automatic biochemical instrument (KHB 450, Shanghai Kehua bio-engineering co., Ltd, Shanghai, China) (31).

### Histology analysis

The ileal tissues were removed and fixed in 4% paraformaldehyde solution, then hematoxylin and eosin were used to stain the paraffin-embedded and cut tissue sections. Light microscopes with computer-assisted morphometric systems were used to measure villus height and crypt depth in each section. The villus height was the distance from the villus tip to the crypt mouth, and crypt depth was the distance from the crypt mouth to the base of the crypt (32, 33).

### Microbiota analysis

DNA extraction and 16S ribosomal RNA amplification were conducted as previously reported (34). Briefly, DNA was extracted from each fecal sample with an E.Z.N.A. ^®^ soil DNA Kit (Omega Biotek, Norcross, GA, USA) according to the standard protocol. The thermal cycling programing was performed as follows: initial denaturation step, 95°C, 3 min; denaturation, 27 cycles, 95°C, 30 s; annealing, 55°C, 30 s; elongation, 72°C, 45 s; and final extension, 72°C, 10 min. The bacterial 16S rRNA was amplified using the universal primers targeting the V3-V4 region 338F/806R and sample sequenced by an Illumina Miseq PE300 platform (Illumina, SD, USA) according to the standard scheme (35). Quality filters were applied to trim raw sequences according to the following criteria: (i) reads with average quality score <20 over a 10-bp sliding window were removed, and truncated reads shorter than 150 bp were discarded. (ii) Truncated reads containing homopolymers longer than 8 nucleotides, more than 0 base in barcode matching, or more than 2 different bases to the primer were removed from the dataset. The possible chimeras were checked and removed via USEARCH using the ChimeraSlayer “gold” database as described by Edgar et al. (36). Operational taxonomic units (OTUs) with 97% similarity cutoff were clustered using USEARCH (37).

### Statistical analyses

Two-tailed Student’s *t*-test was used to compare two groups and ANOVA (one-way analysis of variance) and Tukey’s *post-hoc* analysis was used to compare more than two groups through SPSS 22.0. The data was expressed as the means ± standard errors of the means (SEM). Statistical significance was set at *p* < 0.05.

## Results

### Body weight and organ index

**FIGURE 1:**
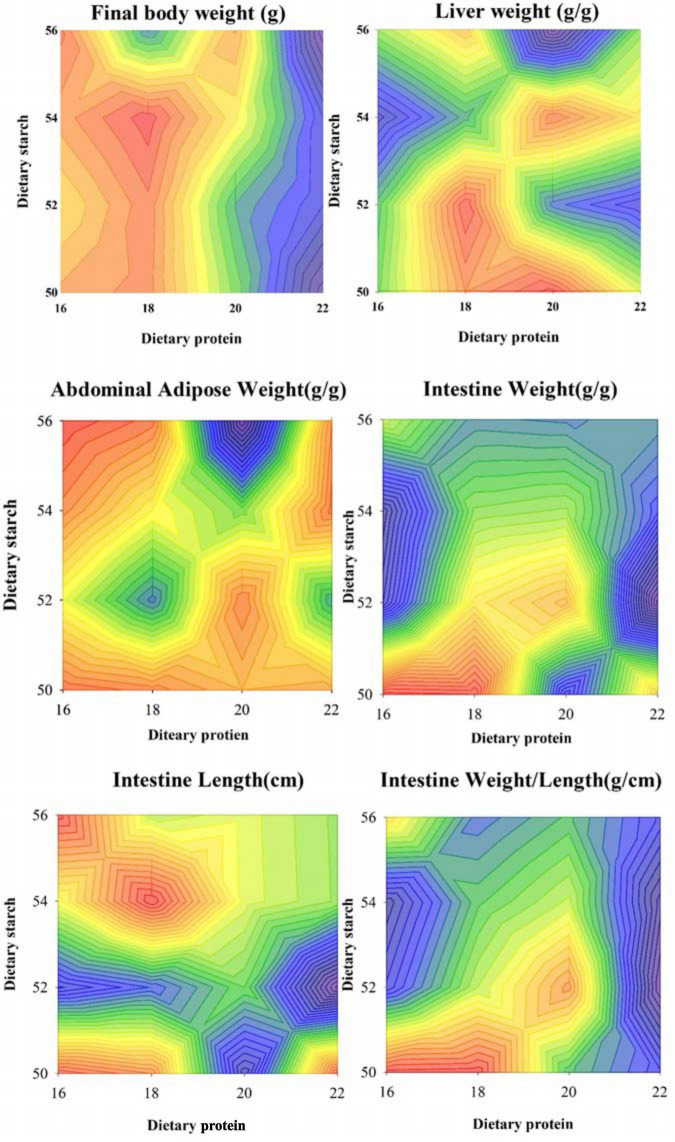
Effects of different ratios of starch and protein in diet on final weight and organ index of mice. The value increases with the deepening of red and decreases with the deepening of blue.

In order to study whether dietary starch and protein can affect the lipid metabolism of the body, we used mice fed diets with different concentrations of starch and protein as models. As shown in Figure 1, the four different ratios of starch had no significant effect on the final body weight of male mice (*p* = 0.307), while the 22% protein group significantly decreased the final body weight of the mice than other protein ratio groups (*p* < 0.05). At the same time, starch and protein had no significant interaction effect on body weight (*p* = 0.246). Different proportions of starch and protein had obvious effects on the liver index of mice, and there was a significant interaction between starch and protein on the liver index (*p* = 0.005). Although different ratios of starch and protein had no significant effect on abdominal adipose weight in mice separately, there was a significant interaction between different starch and protein ratios on abdominal adipose weight (*p* < 0.001). Different proportions of starch have significant effect on the weight of the small intestine of male mice (*p* = 0.005), and the different protein level diet fed the male mice has a very significant difference in the weight of the small intestine (*p* = 0.001). The small intestine weight of mice with 18% protein in the diet was significantly higher than that of other diet groups with 3 protein ratios (*p* < 0.05). In addition, the small intestine of male mice fed the 50% starch diet was much heavier than the 52, 54, and 56% starch diets (*p* < 0.05). Correspondingly, there was a significant interaction between different starch and protein ratios on intestinal weight (*p* < 0.001). Diets with different protein levels had no effect on the length of the small intestine of the mice (*p* = 0.435), however, the 52% starch diet significantly reduced the small intestine length of mice compared with other starch ratio diets (*p* = 0.018). At the same time, the ratio of starch and protein had no significant interaction on the length of the small intestine (*p* > 0.05). Ultimately, different levels of protein had a significant effect on the ratio of small intestine weight to length (*p* < 0.05), but different levels of starch had no such effect. There was a significant interaction between starch and protein on the ratio of small intestine weight to length (*p* < 0.05).

### Influence of the interaction between protein and starch on serum biochemical indexes

**FIGURE 2:**
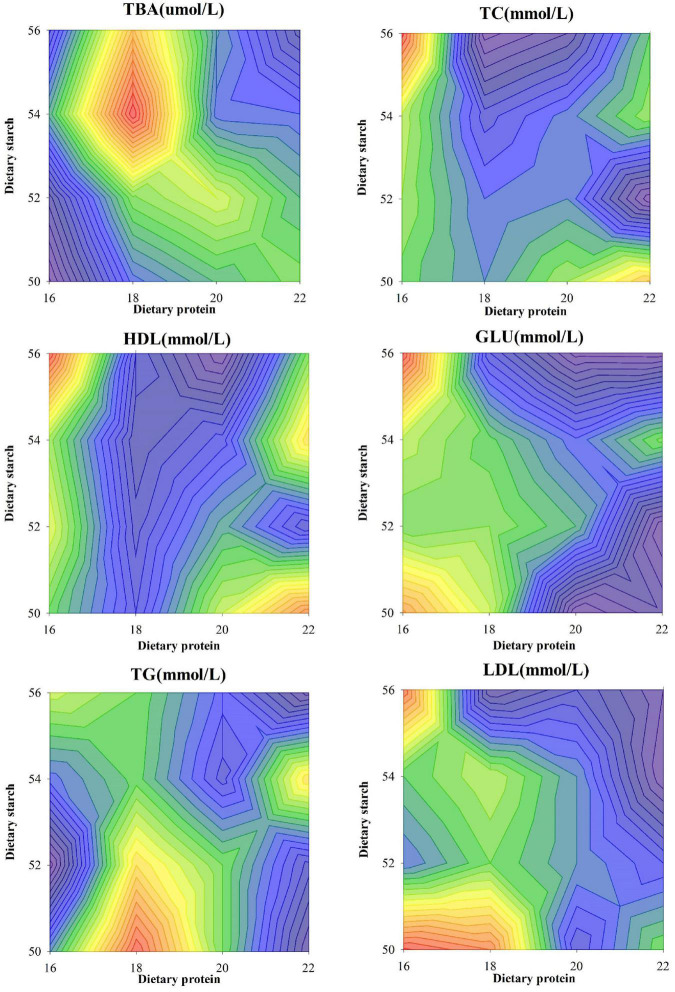
Effects of different ratios of starch and protein in diet on serum lipid levels in mice. The value increases with the deepening of red and decreases with the deepening of blue.

Variation of biochemical index in the serum of male mice was shown in Figure 2. Compared with other protein ratio diets, 18% protein diet significantly increased the serum TBA concentration of mice (*p* < 0.001), and different starch ratio diets had no effect on serum TBA concentration (*p* = 0.442). Compared with the 18 and 20% protein diets, the 16% protein diet significantly increased the serum TC concentration in mice (*p* < 0.05), and the different starch ratio diets had no effect on the serum TC concentration (*p* = 0.301). There was a significant interaction between diets with different starch and protein levels on serum TC in mice (*p* = 0.004). Compared with the 18 and 20% protein diets, the 16 and 22% protein diets significantly increased the serum HDL concentration in mice (*p* < 0.05), and the different starch ratio diets had no effect on the serum HDL concentration (*p* > 0.05). There was a significant interaction between diets with different starch and protein levels on serum HDL in mice (*p* = 0.012), and the combination of 16% protein and 56% starch diets increased serum HDL concentrations in mice. Compared with the 20 and 22% protein diets, the 16% protein diet significantly increased the serum GLU concentration of mice (*p* < 0.05), and the different starch ratio diets had no effect on the serum GLU concentration (*p* > 0.05). There was a significant interaction between diets with different starch and protein levels on the serum GLU of mice (*p* = 0.003), and the GLU concentration in serum was highest in mice fed a combination of 16% protein and 56% starch than other protein and starch diets. There was a significant interaction between diets with different starch and protein levels on serum TG in mice (*p* = 0.003), and the TG concentration in serum of mice fed with 18% protein and 50% starch diets was significantly higher than that of 22% protein and 50, 52, 56% starch diet combination (*p* < 0.05). Different starch ratio diets had no effect on the serum TG concentration of mice (*p* = 0.714). During the experiment, the serum LDL concentration of mice in each group was also affected by diets with different protein levels (*p* = 0.006), but not affected by different starch ratio diets (*p* = 0.854). Diets with different starch and protein levels also had a significant interaction on serum LDL in mice (*p* < 0.001). The LDL concentration in the serum of mice fed the 16% protein and 56% starch diet was significantly higher than that of the 22% protein and 52, 54, and 56% starch diets (*p* < 0.05).

### Intestinal histomorphology analysis

**FIGURE 3:**
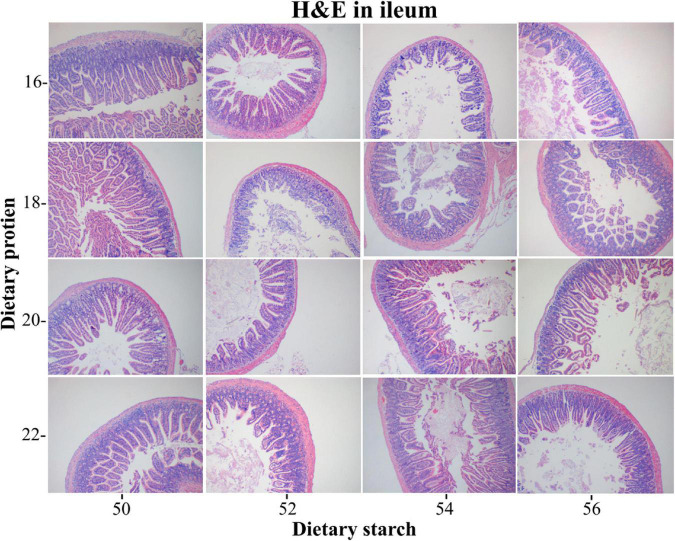
Light microscopy cross-section of ileal tissue by different ratios of starch and protein in diet.

**FIGURE 4:**
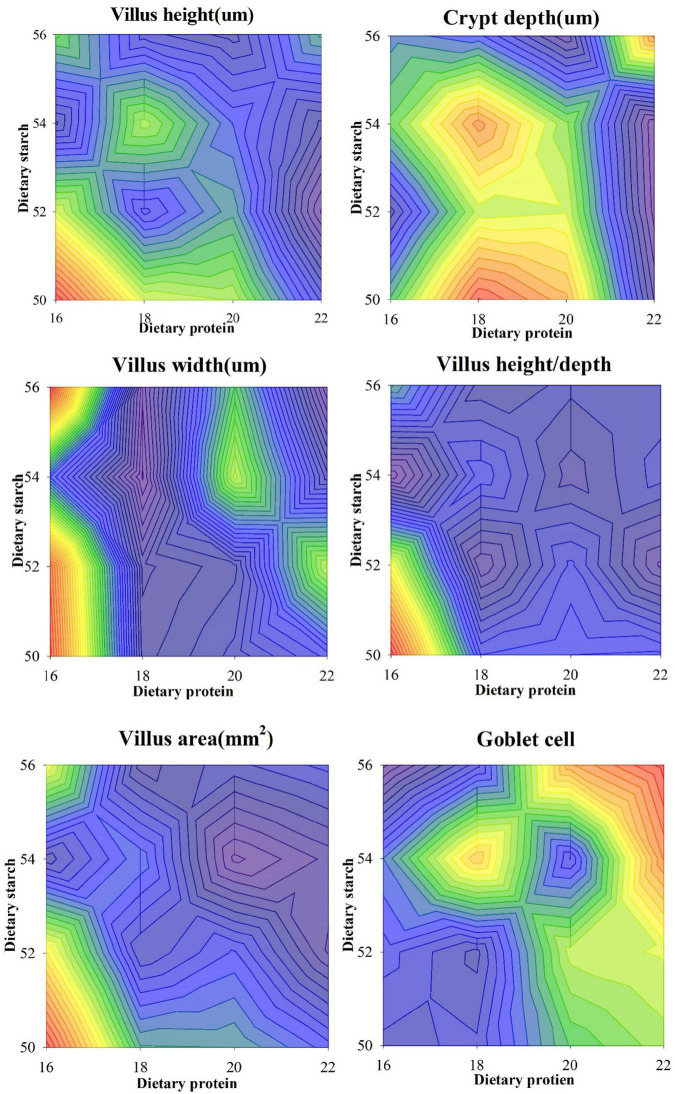
Effects of different ratios of starch and protein in diet on the structure of ileal epithelium in mice. The value increases with the deepening of red and decreases with the deepening of blue.

The ileal tissue morphology under different starch and protein treatments was shown in Figure 3. It was proved from the pictures of ileal tissue HE staining that the low protein diet and the low starch diet were more favorable. The measurement results for ileal tissue were shown in Figure 4, the ileal villus height in the diet tended to decrease with the increase of protein concentration (*p* = 0.059), and different proportions of starch had no effect on the ileal villus height (*p* > 0.05). Different levels of protein and starch also had significant interaction effects on ileal villus height (*p* = 0.044). Diets with different protein and starch ratios had no significant effect on ileal crypt depth in male mice (*p* > 0.05), and there was no interaction effect (*p* > 0.05). The ileal villus width was not affected by the dietary starch ratio (*p* = 0.461); however, the 22% protein diet significantly increased the ileal villus width compared with the other protein ratio diets (*p* < 0.05), and finally different starch and protein ratios had no interaction effect on ileal villus width (*p* = 0.114). The ratio of ileal villus height to crypt depth in mice was affected by different dietary protein and starch content, and the ratio of ileal villus height to crypt depth in mice with 16% protein diet was significantly higher than that of 18, 20, and 22% protein diet (*p* = 0.009). The ratio of villus height to crypt depth was significantly higher in the 50% starch diet than in male mice fed the 54% starch diet (*p* < 0.05). Different dietary protein and starch contents had significant interaction effects on the ratio of ileal villus height to crypt depth in mice (*p* = 0.006). The effects of different dietary protein and starch contents on mice ileal villus area were similar to the effects on the ratio of villus height and crypt depth. And the ratio of ileal villus area in mice with 16% protein diet was significantly higher than other protein ratio diets (*p* = 0.001). The ratio of villus height to crypt depth was significantly higher in the 50% starch diet than in male mice fed the 54% starch diet (*p* < 0.05). The number of goblet cells in the ileum of mice increased significantly with increasing dietary protein content (*p* < 0.05), while the number of ileal goblet cells was not affected by dietary starch content (*p* > 0.05). Different dietary protein and starch levels had significant interaction effects on the ileal number of goblet cells in mice (*p* = 0.007).

### Fecal bacterial diversity and similarity

**FIGURE 5:**
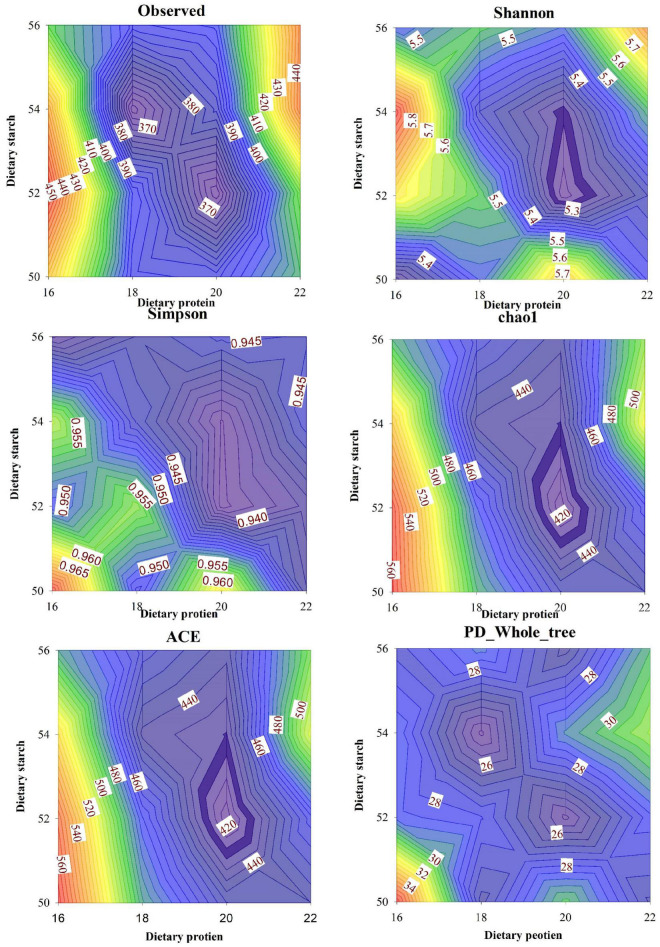
Effects of different ratios of starch and protein in diet on α diversity in the fecal microbiota of mice. The value increases with the deepening of red and decreases with the deepening of blue.

The results of different protein and starch diets on the fecal microbial α-diversity of male mice were shown in Figure 5. The observed species in feces of mice was affected by different dietary protein and starch content, the 16% protein diet significantly increased observed species in the feces compared to other protein levels (*p* < 0.05), and the observed species was significantly higher in mice with 50% starch diet than in mice with 52% starch diet (*p* < 0.05). Different dietary protein and starch contents had significant interaction effects on the observed species in feces of mice (*p* < 0.01). Diet with 16% protein fed to mice significantly increased Shannon index in feces than other different protein level diet (*p* < 0.05), and 50% starch diet fed to mice also significantly increased Shannon index in feces than other starch protein level diet (*p* < 0.05). Different dietary protein and starch levels had significant interaction effects on Shannon index in mice (*p* < 0.01). For the Simpson index in fecal microbiota, the 16% protein diet fed to mice significantly improved the Simpson index in the feces compared to the other protein concentration diets (*p* < 0.05), and the 50% starch diet fed to mice also significantly improved the Simpson index in the feces compared to the other starch concentration diets (*p* < 0.05). Dietary protein and starch concentrations had a significant interaction effect on the Simpson index in mouse feces (*p* < 0.05). The effects of diets with different protein and different starch concentrations on the chao1 index of mouse feces were similar to the Simpson index, both the 16% protein diet and 50% starch diet fed to mice significantly increased the chao1 index than other groups in mouse feces (*p* < 0.05), and dietary protein and starch concentrations had a very significant interaction effect on the chao1 index in mouse feces (*p* < 0.05). The ACE index in feces of mice was affected by different dietary protein and starch content, and the ACE index in feces with 16% protein diet was significantly higher than that of 18, 20, and 22% protein diet (*p* < 0.01). The ACE index was significantly lower in the 52% starch diet than in male mice fed the 50, 54, and 56% starch diet (*p* < 0.05). Different dietary protein and starch contents had a very significant interaction effect on the ACE index in feces of mice (*p* < 0.001). The 16% protein diet significantly increased PD_whole_tree in the feces compared to 18% protein levels (*p* < 0.05), and the PD_whole_tree was significantly higher in mice with 50% starch diet than in mice with 52% starch diet (*p* < 0.05). Different dietary protein and starch contents had a significant interaction effect on the PD_whole_tree in feces of mice (*p* < 0.01).

### Intestinal bacterial community structure

Variation of phylum-level composition (>1%) on the fecal bacterial community of mice fed different starch and protein ratio diets was shown in Table 2, *Bacteroidetes*, *Firmicutes*, *Verrucomicrobia* and *Proteobacteria* were dominant phyla in the feces of mice, accounting for more than 95% of the fecal total bacterial community. Compared with 16 and 22% protein ratio diets, both 18 and 20% protein diets significantly increased the *Bacteroidetes* abundance in feces of mice (*p* < 0.05), and 52% starch ratio diet significantly increased the *Bacteroidetes* abundance than other starch ratio diets of mice (*p* < 0.05). There was a significant interaction between diets with different starch and protein levels on *Bacteroidetes* abundance in feces of mice (*p* < 0.001). Compared with the 18, 20, and 22% protein diets, the 16% protein diet significantly increased the *Firmicutes* abundance in mice (*p* < 0.05), and the 50% starch ratio diet significantly increased the *Firmicutes* abundance than 52 and 56% starch diet in feces of mice (*p* < 0.05). There was a significant interaction between diets with different starch and protein levels on *Firmicutes* abundance in mice (*p* < 0.001). Although *Verrucomicrobia* of phyla proportion varied with the different starch level added to the diet, there was no significant difference on *Verrucomicrobia* abundance in the feces of different starch level (*p* = 0.045). Both of the 16 and 18% protein diets fed to mice decreased *Verrucomicrobia* abundance in feces than 20 and 22% protein diets (*p* < 0.05). Different dietary protein and starch contents had a significant interaction effect on the *Verrucomicrobia* abundance in feces of mice (*p* < 0.001). Compared with the 18 and 22% protein diets, the 16% protein diet significantly decreased the *Proteobacteria* abundance in feces of mice (*p* < 0.05), and the 52% starch ratio diet fed to mice increased *Proteobacteria* abundance significantly in feces than other starch ratio diets (*p* < 0.05). There was a significant interaction between diets with different starch and protein levels on *Proteobacteria* abundance in mice (*p* < 0.001).

**TABLE 2:** Phylum-level composition (>1%) on the fecal bacterial community of mice fed different starch and protein ratio diets.

Diets		*Bacteroidetes*	*Firmicutes*	*unidentified_Bacteria*	*Verrucomicrobia*	*Proteobacteria*
CP	TS					
16	50	50.9	37.5	5.90	3.30	2.12
16	52	58.0	23.5	1.10	14.5	2.69
16	54	58.8	30.5	6.05	1.68	2.74
16	56	75.3	13.9	5.37	1.14	3.88
18	50	70.6	14.1	3.66	4.79	3.98
18	52	77.5	10.2	3.92	1.64	6.28
18	54	81.2	11.3	1.76	1.14	3.76
18	56	70.5	17.8	3.33	1.82	3.71
20	50	68.2	22.9	2.44	2.40	3.24
20	52	80.4	7.60	1.11	5.29	5.41
20	54	68.2	13.9	0.81	13.5	2.05
20	56	69.4	14.5	2.60	11.0	1.80
22	50	72.0	14.5	6.41	3.86	2.19
22	52	69.9	14.2	4.29	5.64	5.49
22	54	61.5	17.4	7.14	8.41	4.95
22	56	55.0	22.1	11.4	8.83	2.20
**Univariate analysis**						
16	–	61.1^b^	27.6^a^	5.63^a^	2.49^b^	2.87^c^
18	–	74.3^a^	14.0^b^	3.00^b^	2.65^b^	4.00^a^
20	–	71.3^a^	15.2^b^	1.89^b^	7.73^a^	3.16^bc^
22	–	64.7^b^	17.0^b^	7.27^a^	6.65^a^	3.74^ab^
–	50	65.4^b^	22.5^a^	4.60^ab^	3.59	2.90^b^
–	52	74.3^a^	11.6^c^	2.82^b^	5.54	5.40^a^
–	54	66.9^b^	18.8^ab^	4.36^ab^	5.72	3.52^b^
–	56	67.5^b^	17.0^b^	5.64^a^	6.06	2.88^b^
SEM		0.010	0.009	0.005	0.005	0.002
CP *P*-value		<0.001	<0.001	<0.001	<0.001	0.007
TS *P*-value		0.360	0.006	0.200	0.045	0.001
CP × TS *P*-value		<0.001	<0.001	0.457	<0.001	<0.001

CP, crude protein; TS, starch; Each group contains 10 samples; SEM, standard error of the mean. In the same column, values with no letter or the same letter superscripts mean no significant difference (*p* > 0.05), while with different small letter superscripts mean significant difference (*p* < 0.05).

Downward to genus levels, *Helicobacter*, *Alloprevotella*, *Akkermansia, Parasutterella*, and *Bacteroides* were the predominant genera in feces of sixteen groups. As shown in Table 3, the different starch ratio diets had no effect on the relative abundance of *Akkermansia* and *Bacteroides* in feces of mice (*p* > 0.05). But 20 and 22% protein diets significantly increased the *Akkermansia* abundance than 16 and 18% protein diets fed to mice (*p* < 0.05), and 18% protein diet significantly increased the *Bacteroides* abundance than other three protein diets fed to mice (*p* < 0.05). Diets with different starch and protein levels had a very significant interaction on the relative abundance of *Akkermansia* (*p* < 0.001) and *Bacteroides* in feces of mice (*p* = 0.001). The different starch diets did not change significantly the *Helicobacter* abundance in mice (*p* = 0.360), but 22% protein diet significantly increased the *Helicobacter* abundance than 18 and 20% protein diets fed to mice (*p* < 0.05). There was no significant interaction between diets with different starch and protein levels on *Helicobacter* abundance in mice (*p* = 0.483). Compared with the 18 and 20% protein diets, the 16% protein diet significantly increased the relative abundance of *unidentified_Lachnospiraceae* and *unidentified_Ruminococcaceae* in feces of mice (*p* < 0.05), and the 50% starch ratio diet significantly increased the *unidentified_Lachnospiraceae* and *unidentified_Ruminococcaceae* abundance than 52% starch fed to mice in feces (*p* < 0.05). There was a very significant interaction between diets with different starch and protein levels on the *unidentified_Lachnospiraceae* abundance (*p* = 0.012) and *unidentified_Ruminococcaceae* abundance of mice (*p* = 0.001). Compared with 16 and 18% protein ratio diets, both 20 and 22% protein diets significantly decreased the *Parabacteroides* and *Alistipes* abundance in feces of mice (*p* < 0.05), and 52% starch ratio diet significantly decreased the *Parabacteroides* and *Alistipes* abundance than 50% starch ratio diet of mice (*p* < 0.05). There was a very significant interaction between diets with different starch and protein levels on *Parabacteroides* (*p* = 0.014) and *Alistipes* (*p* = 0.001) abundance in feces of mice. The *Alloprevotella* abundance in feces was highest to mice fed a combination of 16% protein and 56% starch than other protein and starch diets fed to mice. There was a significant interaction between diets with different starch and protein levels on *Alloprevotella* abundance in mice (*p* < 0.001). The *Parasutterella* abundance in feces of mice fed with 22% protein and 56% starch diets was lowest than other protein and starch diets fed to mice. Different starch or protein ratio diets had effect on the *Parasutterella* abundance of mice and a very significant interaction between diets with different starch and protein levels on *Parasutterella* abundance in feces of mice (*p* = 0.004).

**TABLE 3:** Genus-level composition (>1%) on the fecal bacterial community of mice fed different starch and protein ratio diets.

Diets		*Helicobacter*	*Alloprevotella*	*Akkermansia*	*unidentified_ Lachnospiraceae*	*Parasutterella*	*Parabacteroides*	*Bacteroides*	*unidentified_ Ruminococcaceae*	*Alistipes*
CP	TS									
16	50	5.85	2.33	3.30	4.33	0.69	0.71	0.94	3.00	1.05
16	52	1.09	4.47	14.50	2.92	2.04	0.50	0.47	1.20	1.63
16	54	6.02	6.07	1.68	3.36	1.28	1.53	1.83	2.01	1.13
16	56	5.28	14.37	1.14	0.95	2.85	1.42	1.32	2.13	1.11
18	50	3.60	3.41	4.79	1.34	3.46	1.88	0.95	2.20	1.28
18	52	3.86	4.99	1.64	1.33	5.47	0.62	1.25	1.48	0.70
18	54	1.71	11.61	1.14	1.03	2.64	1.23	2.13	1.30	0.47
18	56	3.23	2.90	1.82	2.39	2.89	0.68	2.62	1.86	1.12
20	50	2.39	4.74	2.40	2.74	2.19	0.73	1.84	2.33	1.08
20	52	1.01	8.33	5.29	0.89	5.01	0.26	1.29	0.92	0.31
20	54	0.77	3.38	13.53	3.04	2.20	0.44	0.82	1.02	0.42
20	56	2.58	5.52	10.96	2.03	1.56	0.36	0.89	0.99	0.74
22	50	6.37	4.91	3.86	2.94	1.62	0.36	1.00	1.04	0.24
22	52	4.26	3.84	5.64	2.27	3.56	0.45	1.17	1.11	0.40
22	54	7.11	6.21	8.41	2.72	2.28	0.91	1.34	1.11	0.58
22	56	11.22	2.56	8.83	3.13	0.31	0.46	0.45	1.10	0.81
**Univariate analysis**										
16	–	5.58^a^	7.26^a^	2.49^b^	2.97^a^	1.62^c^	1.19^a^	1.34^b^	1.76^b^	1.12^a^
18	–	2.93^b^	5.93^ab^	2.65^b^	1.54^b^	3.21^a^	1.27^a^	1.83^a^	1.39^c^	0.96^a^
20	–	1.83^b^	5.57^ab^	7.73^a^	2.16^b^	2.66^ab^	0.46^b^	1.22^b^	1.09^d^	0.68^b^
22	–	7.21^a^	4.44^b^	6.65^a^	2.77^a^	1.95^bc^	0.55^b^	1.01^b^	2.37^a^	0.50^b^
–	50	4.55^ab^	3.85^b^	3.59	2.84^a^	2.04^b^	0.92^a^	1.17	1.48^b^	0.92^a^
–	52	2.76^b^	5.78^ab^	5.54	1.66^b^	4.25^a^	0.39^b^	1.19	1.09^c^	0.46^b^
–	54	4.32^ab^	7.07^a^	5.72	2.54^ab^	2.08^b^	1.06^a^	1.57	1.38^b^	0.68^b^
–	56	5.56^a^	6.41^a^	6.06	2.15^ab^	1.87^b^	0.72^ab^	1.29	2.14^a^	0.93^a^
SEM		0.005	0.005	0.005	0.002	0.002	0.001	0.001	0.005	<0.001
CP *P*-value		<0.001	0.302	<0.001	0.038	<0.001	0.004	0.032	<0.001	<0.001
TS *P*-value		0.360	0.015	0.050	0.211	0.001	0.121	0.284	<0.001	0.024
CP × TS *P*-value		0.483	<0.001	<0.001	0.012	0.004	0.014	0.001	0.001	0.001

CP, crude protein; TS, starch; Each group contains 10 samples; SEM, standard error of the mean. In the same column, values with no letter or the same letter superscripts mean no significant difference (*p* > 0.05), while with different small letter superscripts mean significant difference (*p* < 0.05).

## Discussion

Interactions between starch and protein have been increasingly appreciated during the last few years, for example in combined system of protein and starch for formulation of novel functional foods such as bakery products, infant foods, dessert and snack foods (4, 38–40), owing to their rich functionality, superior nutritional value and significant bioactivity. In this study, male mice were used as an experimental model to investigate the effects of different levels of protein and starch diets on fat deposition, fecal microbiota, and small intestinal tissue structure in mice. Studies have shown that dietary protein and starch played a role in regulating lipid biological synthesis.

The high protein diet reduced liver fat more effectively than the low protein diet (41). For example, in mice and human research, the high protein diet has proven to increase energy consumption, reduce blood glucose levels, promote fat oxidation, thereby supporting weight loss, and reducing liver fat (42–45). The relatively lower carbohydrate content in the high protein diet may also be part of the cause of new fat production and reduced intrahepatic fat. In addition, compared with fat-generating genes, in several rats’ research, protein intake in diet has not changed the expression of genes related to lipid oxidation or substrate oxidation (46, 47). Our results showed that the 22% protein group significantly decreased the final body weight of the mice than other protein ratio groups, and 20% protein with 56% starch fed to mice got the lowest abdominal adipose weight than others group. The reason may be that the fat intake and lipid biological synthesis may be inhibited. The liver is one of the important organs for host lipid metabolism, including lipid assembly and transportation (48). Meanwhile, the absorption and transport of lipids in the small intestine also determines the fate of lipid metabolism (49), and a two-way effect occurs with intestinal microbes and metabolites (50, 51). In this study, different starch and protein diets had less effect on the final liver and abdominal adipose weight in male mice, which may also be due to the shorter experimental period.

The biochemical indicators of the blood could not only feedback the health of the host and the strength of immune function, but also revealed the biological characteristics of different hosts (52). Jenkins et al. (53) studied the effect of the starch–protein complex interaction in wheat-based food, founding that the presence of protein in white flour produced a reduction in the digestibility rate of starch *in vitro* and had a direct relationship with the decrease of the glycemic response *in vivo*, which may be relevant in the effect of food on the gastrointestinal tract. In the present study, feeding male mice with a 22% high-protein diet reduced blood Glu level, while starch ratio had no significant effect on Glu concentrations. This showed that the Glu level in blood was easily affected by the protein in the diet, and the Glu concentration in the blood increased with the lower the protein content. The study demonstrated that plasma lipoprotein, especially HDL, can be combined with LPS and preferentially shunt liver cells away from liver macrophages, thereby increasing LPS excretion through the bile and preventing immune responses (54). When the level of TC in the host’s blood rises, hypertrophymia will occur. Compared with LDL, HDL level may lead to this situation (55). Some researchers have compared the influence of high and low diets on obesity and their related diseases. One of the main discoveries in the system summary was that the HP solution had a favorable impact on TG and HDL (56). Our study confirmed that the protein in the dietary formula affected the indicators of blood lipid metabolism in mice, and these indicators were hardly affected by the proportion of starch in the diet. These data were very important for preventing hyperlipidemia and heart and liver disease (57).

Dietary nutrients can modulate the small intestinal tissue morphology and digestive function of animals, and the intestinal barrier function was very important to the host (58–60). Villus height and crypt depth are important indicators to measure the digestion and absorption function of the small intestine. The depth of the crypts reflects the rate of cell formation, while shallower crypts indicate an increased rate of cell maturation and enhanced secretory function. The height of villi and the depth of crypts can comprehensively reflect the functional status of the small intestine (34, 61). In our study, the ileal crypt depth was not affected significantly by the ratio of dietary protein and starch. Since the intestinal surface area represented by the tight packing and long projections of villi showed the maximal absorption of nutrients allowed (62), the decreased ratio of villi height to crypt depth in the ileum of the 22% CP group showed reduced nutrient absorption. In the present study, although the increased dietary protein concentration did not significantly alter the villus height and crypt depth of the small intestine, the ratio of villus height to crypt depth decreased, and the villus area was also reduced, so the high concentration of protein in the diet impaired the mucosal morphology of the small intestine resulting in underdevelopment of the small intestine, and dietary starch has a negligible effect on intestinal morphology.

The integrity of the intestinal structure and dynamic balance of intestinal microbiota guarantee the chemical induction and digestive functions of the gut, which is the premise for nutrient absorption, metabolism, and deposition. With the development of gene sequencing technology, we can explore the impact of changes in animal diet on the structure and function of intestinal microorganisms (63, 64). The *Bacteroidetes* and *Firmicutes* were dominant phyla in the feces of mice in this study. The decline of *Streptococcus* and *Escherichia-Shigella* in the ileum when dietary protein concentration decreased by 3 percentage points suggested the positive effect (65). In this study, the abundance of *Streptococcus* and *Escherichia-Shigella* was less than 1%, both of them responsed to the diet were negligible. Recently, altered gut microbiome has been shown to be associated with host lipid metabolism through dietary structure (49, 66, 67). Germ-free mice demonstrated that the changes of the gut microbiota community including decreased relative abundance of *Lachnospiraceae* and an enhanced occurrence of *Desulfovibrionaceae*, *Clostridium lactatifermentans* and *Flintibacter butyricus* were associated with impaired glucose metabolism, lowered counts of enteroendocrine cells, fatty liver, and elevated amounts of hepatic triglycerides, cholesteryl esters, and monounsaturated fatty acids by high-fat diet (68). In the present study, the content of *unidentified_Lachnospiraceae* in mouse feces was higher and affected by the interaction between protein and starch in the diet, and the effect of protein was greater than that of starch. However, the content of *Desulfovibrionaceae*, *Clostridium lactatifermentans* and *Flintibacter butyricus* in mouse feces was very low, so it was not investigated. Furthermore, gut microbiota-mediated cholesterol metabolism via a microbial cholesterol dehydrogenase played an important role in host cholesterol homeostasis (69). Interestingly, there may be a bridge between gut microbes and metabolites and lipid levels, and several relevant genes regulated the concentration of lipids. A study has found that theabrownin reduced liver cholesterol and decreased lipogenesis by the gut microbe-bile acid-FXR-FGF15 signaling pathway (50). In our study, the concentration of TBA in blood was the highest when the diet contained 18% protein, and the ratio of dietary starch had no significant effect on TBA. Therefore, dietary protein may regulate host lipid metabolism through some bacteria, which required more follow-up research.

Futher, the microbial composition, along with a wide range of microbial metabolites, played a complex role in various host processes, such as resistance to autoimmunity (70). For the ileal microbiota in pigs, when dietary protein dropped by 3 percentage points, the decreased *Enterobacteriaceae* within the *Proteobacteria* phyla has been considered to contain many pathogenic bacteria (71), indicating the potential for inhibiting pathogens with moderate dietary protein restriction. In general, the abundance of *Proteobacteria* in mouse feces was the highest when the diet contained 56% starch in this study. Therefore, the increase of dietary starch content was harmful to the health of mice from the perspective of microorganisms. In the colon, when dietary protein concentration declined, the abundance of *Firmicutes* increased while *Bacteroidetes*, *Spirochaetae*, and *Verrucomicrobia* decreased (65). Consistent with previous research, the abundance of *Firmicutes* in mouse feces decreased with the increase of dietary protein, while the abundance of *Bacteroides* and *Verrucomicrobia* decreased conversely in this study. Since microorganisms were greatly affected by dietary formulation, starch and protein had obvious interaction effect on the main dominant bacterial genera, which provided insights for future exploration of the effects of specific microorganisms on dietary formulation.

## Conclusion

In conclusion, our results showed that the 22% protein group significantly decreased the final body weight of the mice than other protein ratio groups, and 20% protein with 56% starch fed to mice got the lowest abdominal adipose weight than others group. The gut microbial abundance was altered by different starch and protein fed to male mice. Our study provided a more comprehensive understanding of mouse fecal microbial responses to different starch and protein diets, including lipid metabolism and gut barrier.

## Data availability statement

This original contributions presented in this study are included in the article/supplementary material, further inquiries can be directed to the corresponding authors.

## Ethics statement

This animal study was reviewed and approved by Hunan Agricultural University Institutional Animal Care and Use Committee (202105). Written informed consent was obtained from the owners for the participation of their animals in this study.

## Author contributions

JM and PZ designed the experiment. PZ supported the funding. JM and KW conducted the experiment. KW, JM, MZ, XG, and YZ collected and analyzed data. KW wrote the manuscript. PZ, JC, and JM revised the manuscript. All authors contributed to the article and approved the submitted version.
